# Chromosome Segregation in Closed Mitosis Under an Excess of Nuclear Envelope

**DOI:** 10.1111/boc.70011

**Published:** 2025-05-20

**Authors:** Noelia Rodríguez‐Herrera, Silvia Santana‐Sosa, Sara Medina‐Suárez, Samantha Morais‐Armas, Emiliano Matos‐Perdomo, Félix Machín

**Affiliations:** ^1^ Unidad de Investigación Hospital Universitario Nuestra Señora de Candelaria Instituto de Investigación Sanitaria de Canarias (IISC) Santa Cruz de Tenerife Spain; ^2^ Instituto de Tecnologías Biomédicas Universidad de La Laguna San Cristóbal de La Laguna Spain; ^3^ Facultad de Ciencias de la Salud Universidad Fernando Pessoa Canarias Las Palmas de Gran Canaria Spain

**Keywords:** chromosome segregation, closed mitosis, Lipin, Nem1, nuclear envelope, Nvj1, rDNA, *Saccharomyces cerevisiae*, vacuole

## Abstract

**Background:**

Two major types of cell division occur in eukaryotic cells regarding the dismantlement or not of the nuclear envelope (NE) in mitosis, open and closed mitosis, respectively. In the budding yeast *Saccharomyces cerevisiae*, the prototypical model for closed mitosis, the Nem1‐Spo7 phosphatase complex, which regulates lipid metabolism, plays a key role in coordinating NE expansion throughout the cell cycle. Indeed, Nem1 depletion leads to abnormal NE evaginations in interphase, which protrude the ribosomal DNA (rDNA) and the nucleolus. However, the specific impact of these NE and chromosome organization abnormalities during chromosome segregation in anaphase remains poorly understood.

**Results:**

Our study investigated chromosome segregation and NE dynamics during closed mitosis in relation to the presence or absence of Nem1. Nem1 was depleted by means of the auxin degron system. Nem1 depletion led to the formation of chromatin protrusions in interphase, particularly at the rDNA locus, as it has been reported before for *nem1* mutants. These protrusions persisted into anaphase and were associated with delayed recoiling of the rDNA‐bearing chromosome XII right arm, resulting in lagging chromatin during late anaphase. Additionally, cells can maintain nucleus‐vacuole junctions (NVJs) during anaphase, suggesting that vacuoles may play a role in shaping NE morphology during chromosome segregation.

**Conclusion:**

Our findings suggest that the Nem1‐Spo7/lipin regulation of the NE size is crucial for the timely segregation of the rDNA‐bearing chromosome during closed mitosis. Thus, the NE homeostasis actively contributes to chromosome segregation and the spatial organization of chromosomes in subsequent cell cycles. In addition, the persistent association between the NE and vacuoles in anaphase further underscores how cumbersome organelle interactions can become during closed mitosis, opening inspiring research avenues.

## Graphical abstract

This study investigated the impact of Nem1‐Spo7/lipin‐mediated nuclear envelope (NE) size regulation during closed mitosis in *Saccharomyces cerevisiae*. Nem1 depletion resulted in persistent chromatin protrusions, particularly at the rDNA locus, leading to lagging chromosome XII's right arm sisters during late anaphase. The research also revealed that nucleus‐vacuole junctions (NVJs) can be maintained during anaphase, suggesting that vacuoles can impinge on chromosome segregation. These findings highlight the crucial role of NE size control in ensuring timely chromosome segregation during closed mitosis.

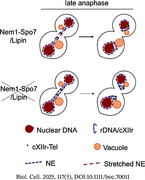

## 1 Introduction

In eukaryotes, from yeast to humans, the interphase nucleus typically adopts a spherical or oval shape. Deviations from these canonical nuclear shapes are frequently associated with cellular dysfunction and severe diseases such as cancer, except in some specialized cell types such as cardiomyocytes and neutrophils (Chow et al. 2012; Skinner and Johnson 2017; Zink et al. 2004). These abnormal nuclear morphologies often arise from an imbalance between the production of nuclear envelope (NE) membranes and the expansion or contraction required to accommodate changes in nuclear or cellular size (Cantwell and Nurse 2019; Deolal et al. 2021). This dynamic regulation of NE size is particularly important in organisms that undergo closed mitosis, in which the NE remains intact throughout chromosome segregation (Arnone et al. 2013; Dey and Baum 2021; Makarova and Oliferenko 2016).

Despite its significance, the mechanisms underlying NE expansion and contraction remain poorly understood. The outer membrane of the NE is continuous with the endoplasmic reticulum (ER), which buffers fluctuations in NE size (Deolal et al. 2024; Kume et al. 2019; Ungricht and Kutay 2017; Zhang and Oliferenko 2013). Additionally, new membranes can be generated or removed directly at the NE to modulate its dimensions (Barbosa et al. 2019). The budding yeast *Saccharomyces cerevisiae* has served as a valuable model for studying nuclear shape and NE regulation (Deolal et al. 2021; Mekhail and Moazed 2010; Santana‐Sosa et al. 2023; Taddei and Gasser 2012). In this organism, which undergoes closed mitosis, NE expansion is a prominent feature of anaphase, coinciding with the elongation of the nucleus along the mitotic spindle axis (Wang et al. 2016). Notably, NE expansion begins in metaphase, preceding the onset of nuclear elongation, as cells arrested in G2/M continue to expand the NE, resulting in deformations such as flares, escapades, lobulations, and toroidal nuclei (Garcia et al. 2022; Hattier et al. 2007; Jorgensen et al. 2007; Matos‐Perdomo et al. 2022; Meseroll and Cohen‐Fix 2016; Wang et al. 2016).

NE expansion in G2/M is driven by the synthesis of new phospholipids from fatty acids, a process regulated through the modulation of lipin (Pah1) activity (Barbosa et al. 2015; Pascual and Carman 2013). Phosphatidic acid, a lipid precursor, is diverted from energy storage lipids toward phospholipid synthesis by downregulating lipin activity (Kwiatek et al. 2020; Pascual and Carman 2013). The regulation of lipin is controlled by phosphorylation; hyperphosphorylated lipins are generally inactive and become dephosphorylated and active via the Nem1‐Spo7 phosphatase complex, particularly under nutrient‐poor conditions sensed by the TORC1 complex (Dubots et al. 2014; Khondker et al. 2022; Kwiatek et al. 2020). Consequently, mutants lacking functional Pah1, Nem1, or Spo7 exhibit excessive NE membrane synthesis, even in the absence of nutrients, leading to enlarged and aberrantly shaped nuclei, reminiscent of the deformations seen during prolonged G2/M arrests (Campbell et al. 2006; Santos‐Rosa et al. 2005; Siniossoglou et al. 1998; Webster et al. 2010).

While the roles of *pah1*, *nem1*, and *spo7* mutants in producing abnormal NE morphology have been well characterized during interphase and G2/M, little is known about how these phenotypes affect late mitosis, particularly anaphase and telophase. This knowledge gap is significant because (i) maintaining NE integrity is crucial for the successful completion of closed mitosis, (ii) NE protrusions are often associated with vacuoles (Hattier et al. 2007; Matos‐Perdomo et al. 2022), potentially influencing organelle redistribution during nuclear elongation, and (iii) NE deformations are not randomly distributed but preferentially localized to regions associated with the nucleolus (Campbell et al. 2006; Male et al. 2020; Matos‐Perdomo et al. 2022; Walters et al. 2014; Witkin et al. 2012). The nucleolus, which specializes in ribosomal biogenesis, contains the ribosomal DNA (rDNA) array, the segregation of which is a unique challenge in yeast. Indeed, rDNA segregation occurs late in anaphase and requires specialized mechanisms that involve the suppression of rDNA transcription by the cell cycle phosphatase Cdc14 as well as TORC1 signaling (Clemente‐Blanco et al. 2009; de los Santos‐Velázquez et al. 2017; Machín et al. 2006; Matos‐Perdomo and Machín 2018). Transcription suppression is in turn required for condensin loading on the locus, which helps resolve catenated rDNA and facilitates the final stages of chromosome compaction, allowing for the successful segregation of the rDNA and the whole right arm of chromosome XII (cXIIr), where the rDNA is housed (D'Amours et al. 2004; Machín et al. 2005).

In this study, we investigated how an enlarged NE, as observed in the absence of Nem1, reorganizes during anaphase and impinges on chromosome segregation, with a particular focus on the cXIIr and the rDNA locus. We also explored the interaction between the NE and vacuoles during this process. Our findings reveal that although Nem1 depletion does not block anaphase progression, it delays the recoiling of the cXIIr. Additionally, our results underscore the importance of vacuole‐NE interactions in modulating NE shape even during anaphase, suggesting that in closed mitosis cells must carefully coordinate the structural connections between the NE and other organelles to ensure timely nuclear division.

## 2 Materials and Methods

### 2.1 Yeast Strains and Experimental Conditions

Yeast strains were all derivatives of a *bar1* YPH499 wild‐type haploid strain (congenic to S228C) with the following basic genotype: *MATa ura3‐52 lys2‐801_amber ade2‐101_ochre trp1*‐Δ*63 his3*‐Δ*200 leu2*‐Δ*1 bar1‐*Δ. The strains are listed in Table S1.

Strains were grown overnight in air orbital incubators at 25°C in either YPD media (10 g/L yeast extract, 20 g/L peptone, and 20 g/L glucose) or SCcshl (6.7 g/L yeast nitrogen base without amino acids, 20 g/L glucose, dropout amino acids, and nucleotides mix according to the CSHL 2005 recipe [Amberg et al. 2005]). To deplete Nem1‐aid*, 1 mM of indole‐3‐acetic acid (IAA; Sigma‐Aldrich, I2886) was added to the media unless stated otherwise.

### 2.2 Fluorescence Microscopy

We used two microscopes; a Leica DMI6000B wide‐field fluorescence microscope equipped with an ultra‐sensitive DFC350 camera and a 63x/1.30 immersion objective; and a Zeiss Axio Observer.Z1/7 equipped with an Axiocam 702 sCMOS camera, the Colibri‐7 LED excitation system, and a Plan‐Apochromat 63x/NA 1.40 Oil M27 DIC objective. In both microscopes, narrow‐band filter cubes for co‐visualization of DAPI, CFP, YFP, and mCherry without emission crosstalk were used. The Zeiss microscope was also set for super‐resolution confocal microscopy (LSM980 with Airyscan 2). In the confocal mode, we used the following laser lines to excite the fluorescent tags: 405 nm for CFP, 514 nm for YFP, and 561 nm for mCherry. The bright field (BF) image was acquired with T‐PMT detectors (pinhole 1 AU) in the confocal mode.

For each field, 10–25 z‐stack focal images (0.16–0.3 µm depth) were collected from log‐phase asynchronous cultures. In general, micrographs were taken from cells fixed with 3.7% w/v formaldehyde (Sigma‐Aldrich, 47608) for 15 min. For DAPI staining, fixed cells were pelleted and 1.5 µL of the pellet was mixed with 1 µL of a 4 µg/mL stock of DAPI on the microscope slide. In the case of MDY‐64 staining, fresh non‐fixed cells were washed once with PBS 1X and MDY‐64 (30 µM stock in PBS) was added directly on the slide (2 µL of pellet plus 1 µL of MDY‐64 stock), incubated for 5 min and visualized in the Airyscan 2 mode. Settings for MDY‐64 visualization was 0.2% for laser 405 nm with a detector master gain of 700; in these conditions, Net1‐eCFP was not detected.

Video‐microscopy was performed as described before (Kumar and Mendoza 2016; Matos‐Perdomo et al. 2022). Briefly, time‐lapse images were acquired on cells immobilized in Nunc Lab‐Tek II coverglass eight‐well chambers pretreated with concanavalin A (ConA). This pretreatment started by adding 50 µL of ConA (1 mg/mL in PBS) to a well, which was then incubated in the dark at 25°C for 20 min before the excess of ConA was washed off twice with SCcshl medium. To adhere cells, log‐phase cell cultures in SCcshl were concentrated and 100 µL of the suspension pipetted in the well and kept at 25°C for 20 min. Non‐attached cells were then washed off with the same medium before starting the live cell imaging in 250 µL of SCcshl with or without 1 mM IAA. When Blue CMAC was added to follow vacuole dynamics, the dye was added to the well together with the cell suspension (100 µM final concentration from a 10 mM stock in DMSO). Typically, a single on focus z plane was captured every 5 min during 2 h to minimize both photobleaching and phototoxicity.

The AF6000 (Leica), Zen Blue (Zeiss), and Fiji‐ImageJ (NIH) software were used for image processing and quantification. 2D maximum projections of the stack of z images are shown in the figures. Individual cells were classified into the different cell cycle stages according to the following criteria: G1, unbudded cells; S/G2 bud size <1/2 of the mother and a round nucleus (as seen by DAPI, Hta2‐mCherry or NE markers); G2/M, bud size >1/2 of the mother and a round nucleus; early anaphase, elongated nucleus across the neck with unresolved cXIIr telomere; late anaphase, binucleated with resolved cXIIr telomere.

Approximately 200 cells were scored for each biological replicate, counting all cells in the corresponding fields to avoid bias. Since cells in anaphase generally represented <15% of the population, overrepresentation of this cell cycle stage was scored up to *N* > 60 for dedicated plots. In the case of Nvj1‐YFP, since NE/NVJ labeling was not seen in the entire population, multiple fields (>300 cells in total) were scored until ∼100 cells with visible Nvj1 (and *N* > 30 for anaphases) were reached.

### 2.3 Growth Curves

For assessment of growth rate and yield, strains were first grown exponentially in either YPD or SCcshl before an inoculum was taken and adjusted to an initial OD_600_ = 0.05 in the corresponding media. IAA was added at the indicated concentrations when required. Three replicates of each culture were aliquoted in a flat‐bottomed 96‐well plate and real‐time growth was measured in a Spark TECAN incubator by reading the OD_600_ every 15 min for 24 h with shaking at 96 rpm (6 mm of orbital amplitude). The mean of the three replicates was calculated to obtain the final growth curves.

### 2.4 Data Representation and Statistics

Error bars usually stand for the standard error of the mean (SEM) of independent biological replicates (*n* = 3). In charts where measurement distributions of single cell analysis are represented, the standard deviation (SD) is shown instead. Graphpad Prism 10 was used for statistical tests. Differences between experimental data points were generally estimated using either the unpaired *t*‐test or one‐way ANOVA; the test used in each specific case is indicated in the figure caption. In piled bar charts, statistical comparisons are not represented as their significance can be visually estimated based on the overlap rule for SEM, that is, a gap between error bars greater than their average length implies *p* < 0.05 (Cumming et al. 2007).

## 3 Results and Discussion

### 3.1 Chromosome XII Segregation Is Largely Unperturbed by Growth Media in Wild‐Type Cells

Our first objective was to determine whether different growth media influence the dynamics of chromosome XII (cXII) segregation during mitosis in wild‐type (WT) *Saccharomyces cerevisiae*. This question is important since nutrient‐rich growth media, such as YPD, are expected to yield higher TORC1 activity as well as having more lipid resources and diversity than synthetic complete media (SC) (Klose et al. 2012; Mahto et al. 2014). Among the different formulations of SC media, we chose that of the Cold Spring Harbor Laboratory 2005 (SCcshl) as it provides a richer recipe of amino acids and nucleotides, and their concentrations are higher than in other recipes, which ensures a TORC1 activity closer to that of YPD (Amberg et al. 2005; Chen et al. 2018). The comparison between these two media is important not only because they are the most often used growth media in yeast research, but also because SC media is required for proper attachment during video‐microscopy (Kumar and Mendoza 2016).

**Figure 1:**
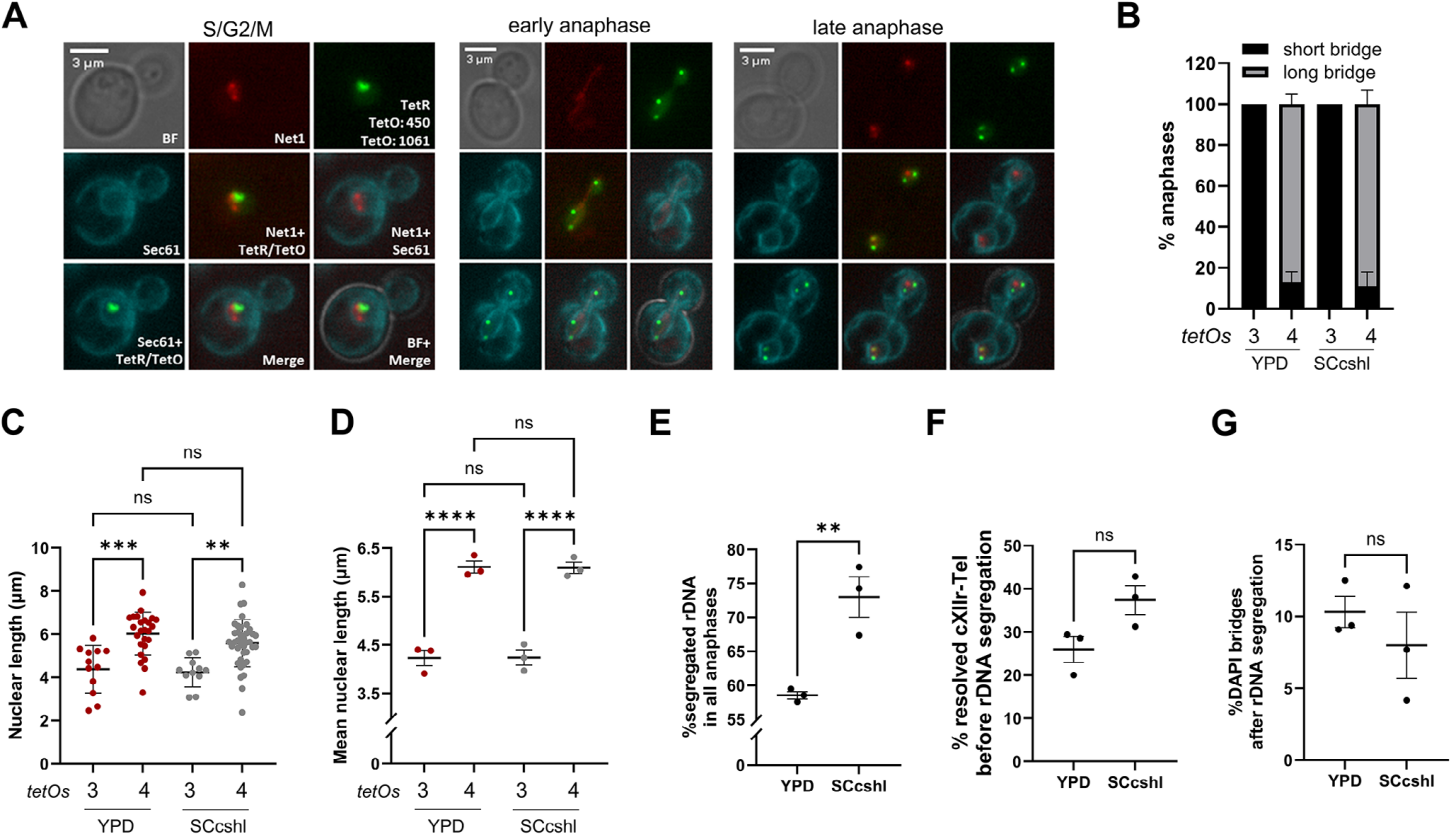
Chromosome XII segregation in wild type cells growing in nutrient rich and complete minimum media. The wild type strain (FM2658) carries the following labels to follow anaphase and chromosome segregation under the fluorescence microscope: Sec61‐eCFP (nuclear envelope), Net1‐mCherry (rDNA), and the TetR‐YFP/*tetOs* system for spotting the centromere‐proximal rDNA flank (*tetO:450*) on the right arm of chromosome XII and its corresponding telomere (*tetO:1061*). The strain was grown to log‐phase at 25°C in the corresponding nutrient‐rich (YPD) or complete minimum (SCcshl) media (mean ± SEM, *n* = 3). Micrographs were taken either directly (A and B) or after fixation in formaldehyde followed by nuclear DNA staining with DAPI (C–G). (A) Representative cells at different cell cycle stages. Early and late anaphase were differentiated based on the morphology and length of Sec61 hourglass NE. (B) The degree of resolution and segregation of the right arm of chromosome XII (cXIIr) was assessed relative to the NE morphology. In early anaphase (short hourglass NE), all cells presented three *tetOs* spots, denoting resolution of *tetO:450* only. In late anaphase (long hourglass NE), nearly 90% of cells had four *tetOs* spots, implying that the whole cXIIr has been resolved. (C) Single cell analysis of nuclear length relative to the cXIIr resolution/segregation status (3 vs. 4 *tetOs*) (mean ± SD). Nuclear length was determined by measuring distal DAPI signals in the anaphase nucleus. Note that full cXIIr resolution associates with a more elongated nucleus, although overlap exists. Plots of a single experiment are shown. (D) Average nuclear length relative to the cXIIr resolution/segregation status (mean ± SEM, *n* = 3). Note that partial and full cXIIr resolution center around nuclear lengths of ∼4.2 and 6.2 µm, respectively. However, there is no difference between the growth media. (E) The degree of rDNA segregation in all anaphases (early plus late). Note that there is a ∼15% increase in the SCcshl medium. (F) The degree of resolution of the cXIIr before rDNA segregation. (G) The degree of DAPI bridges after rDNA segregation. Note that these chromatin bridges are rare in both growth media.

We used a WT strain (FM2658) with fluorescent markers to track the NE (Sec61‐eCFP), the nucleolus/rDNA (Net1‐mCherry), and two loci along the cXIIr. These loci are visualized through the TetR‐YFP/*tetOs* system, and included *tetOs* arrays inserted at cXII coordinates 450 Kb (*tetO:450*; centromere‐proximal rDNA flank) and 1061 Kb (*tetO:1061*; cXIIr telomere). In this strain, growth rate and yield were higher in YPD than in SCcshl (Figure S1A), but we nevertheless observed robust and efficient rDNA and cXIIr segregation under both media (Figure 1). In interphase cells (from G1 to G2/M) taken from a log‐phase asynchronous culture, the NE was round, and there was just one rDNA patch and two green spots, which corresponded to each *tetO* array (Figure 1A; S/G2/M cell). During early anaphase, with the NE in a short hourglass configuration, cXII was partially resolved, as indicated by three *tetOs* spots: two spots corresponding to the resolved rDNA centromere‐proximal regions, and the third spot to the unresolved cXIIr telomere (Figure 1A; early anaphase). In these early anaphase cells, the rDNA was either on one side of the expanding nucleus or forming a bridge across the bud neck. In late anaphase, characterized by full NE elongation, cXIIr resolution was complete in nearly 90% of cells, with four *tetOs* spots indicating that distal segments, including the telomere, had segregated (Figure 1A; late anaphase). Notably, the degree of nuclear elongation closely mirrored the degree of cXII resolution in both growth media (Figures 1B–D and S1B). Thus, the presence of three *tetOs* was associated to shorter hourglass NE bridges and shorter elongation of the DAPI‐stained nuclear mass, whereas the opposite occurred for anaphases with four *tetOs*. As for the degree of rDNA segregation, the SCcshl media led to higher percentages of rDNA segregation in all observed anaphases (Figure 1E), suggesting that rDNA resolution is quicker in SCcshl, a result consistent with less requirements to condense this locus under growth conditions expected to yield less rDNA transcription. Interestingly, in approximately 30% of anaphases, four *tetOs* spots were observed before the rDNA split into two signals (Figures 1F and S1C), indicating that the centromere‐to‐telomere unzipping mechanism for cXIIr resolution, although predominant, is not as prevalent in asynchronous cultures as in synchronized cells (Machín et al. 2005). Likewise, in ∼10% of late anaphase cells with segregated rDNAs, a DAPI bridge was still observed connecting the segregated nuclear masses (Figures 1G and S1D). However, no differences between growth media were noticed for these phenotypes.

### 3.2 Nem1 Depletion Delays Chromosome XII Arm Recoiling After rDNA Segregation

To investigate the role of Nem1 in chromosome segregation, we utilized a nem1‐aid* OsTIR1 strain (FM2748), which allows conditional degradation of Nem1 upon the addition of the auxin indole‐3‐acetic acid (IAA) (Matos‐Perdomo et al. 2022). In order to accommodate the new genetic requirements in this strain, we opted for visualizing only the rDNA and the *tetO:1061* to assess cXIIr segregation. Likewise, we added the Hta2‐mCherry as a reporter of chromatin, so we could also follow chromosome segregation through video‐microscopy. In addition, we included the parental *NEM1 OsTIR1* strain as a control since some phenotypic consequences have been reported after the sole addition of IAA or IAA plus *OsTIR1* (Domeni Zali and Moriel‐Carretero 2023; Nicastro et al. 2021; Prusty et al. 2004).

**Figure 2:**
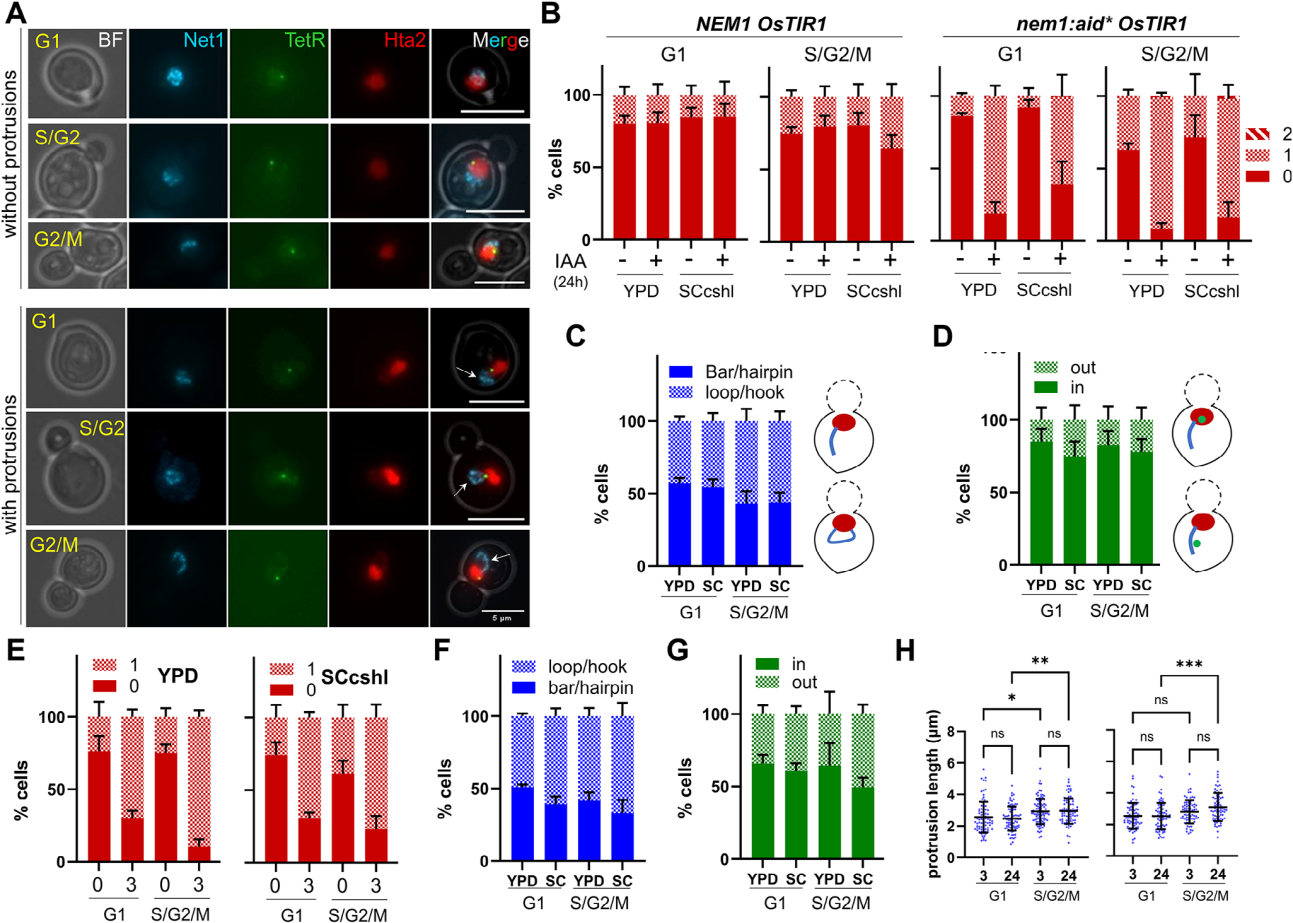
Interphase chromatin protrusions after Nem1 depletion. The strain (FM2748) is isogenic to the one shown in Figure 1, but carries the following labels: Hta2‐mCherry (chromatin), Net1‐eCFP (rDNA), and TetR‐YFP/*tetO:1061* (cXIIr telomere). It also carries the nem1:aid* allele and the OsTIR1 system for depleting Nem1‐aid* after auxin (IAA) addition. (A) Representative micrographs of log‐phase FM2748 cells without and with rDNA chromatin protrusions. Cells in G1, S/G2 and G2/M cells are shown. In all cases, protrusions (white arrows) correspond to the rDNA locus. (B) Quantification of chromatin protrusions relative to cell cycle stages shown in (A), growth media (YPD vs. SCcshl), and − vs. + IAA for 24 h (mean ± SEM, *n* = 3). FM2748 (nem1:aid*) and its wild type (WT) parental FM2707 (as FM2748 but with a wild type *NEM1*) were assessed. Note that depletion of Nem1 causes a single rDNA protrusion in most cases. They are observed in G1 and S/G2/M. (C) Characterization of rDNA shape in the +IAA (24 h) protrusions (mean ± SEM, *n* = 3). (D) Relative location of the cXIIr telomere in the +IAA (24 h) protrusions (mean ± SEM, *n* = 3). (E) As in (B) but before (0 h) and just 3 h after IAA addition in the FM2748 strain (mean ± SEM, *n* = 3). (F) Characterization of the rDNA shape in the +IAA (3 h) protrusions (mean ± SEM, *n* = 3). (G) Relative position of the cXIIr telomere in the +IAA (3 h) protrusions (mean ± SEM, *n* = 3). (H) Protrusion length after 3 and 24 h in IAA (mean ± SD). One way ANOVA followed by the Tukey test was applied for statistical assessment (***p < 0.001; **p < 0.01; *p < 0.05; ns, *p* > 0.05).

Nem1 depletion was rather tolerable for the strain growth as reported from both spot dilution assays and growth curves (Figure S2A,B). The growth curves showed a slightly slower log phase progression after Nem1 depletion, and clonogenic assays after an incubation with IAA for a single cell cycle indicated a probable small decrease in viability (∼20%; Figure S2C). Overnight incubation with 1 mM IAA led to striking nuclear abnormalities during interphase, most notably chromatin protrusions (Figure 2A,B). This phenotype was evident in cells at all interphase stages, from G1 to G2/M, and mostly consisted of a single chromatin protrusion (as seen by Hta2) that predominantly localized to the rDNA region (as seen by Net1). These chromatin protrusions were only seen in the nem1‐aid* strain, disregarding an IAA off‐target effect, and likely stem from the abnormal NE expansion described before for the *nem1‐spo7* mutants (Campbell et al. 2006). Unlike previous observations though, we found the protrusion in G1 cells as well. The rDNA in the protrusion acquired different visible shapes that we grouped as either bars (one‐way) or loops/hooks (round‐trip), with the possibility that some bars are actually hairpins (hence round‐trip) that we could not resolve under the microscope (Figure 2C). Regardless, bars and loops represented around 50% each in both growth media and interphase stages. The cXIIr‐Tel green spot further allowed us to conclude that most of the protruding rDNA bars were genuine, that is, one‐way, with 80% of spots localizing in the bar or near the tip (Figure 2D).

**Figure 3:**
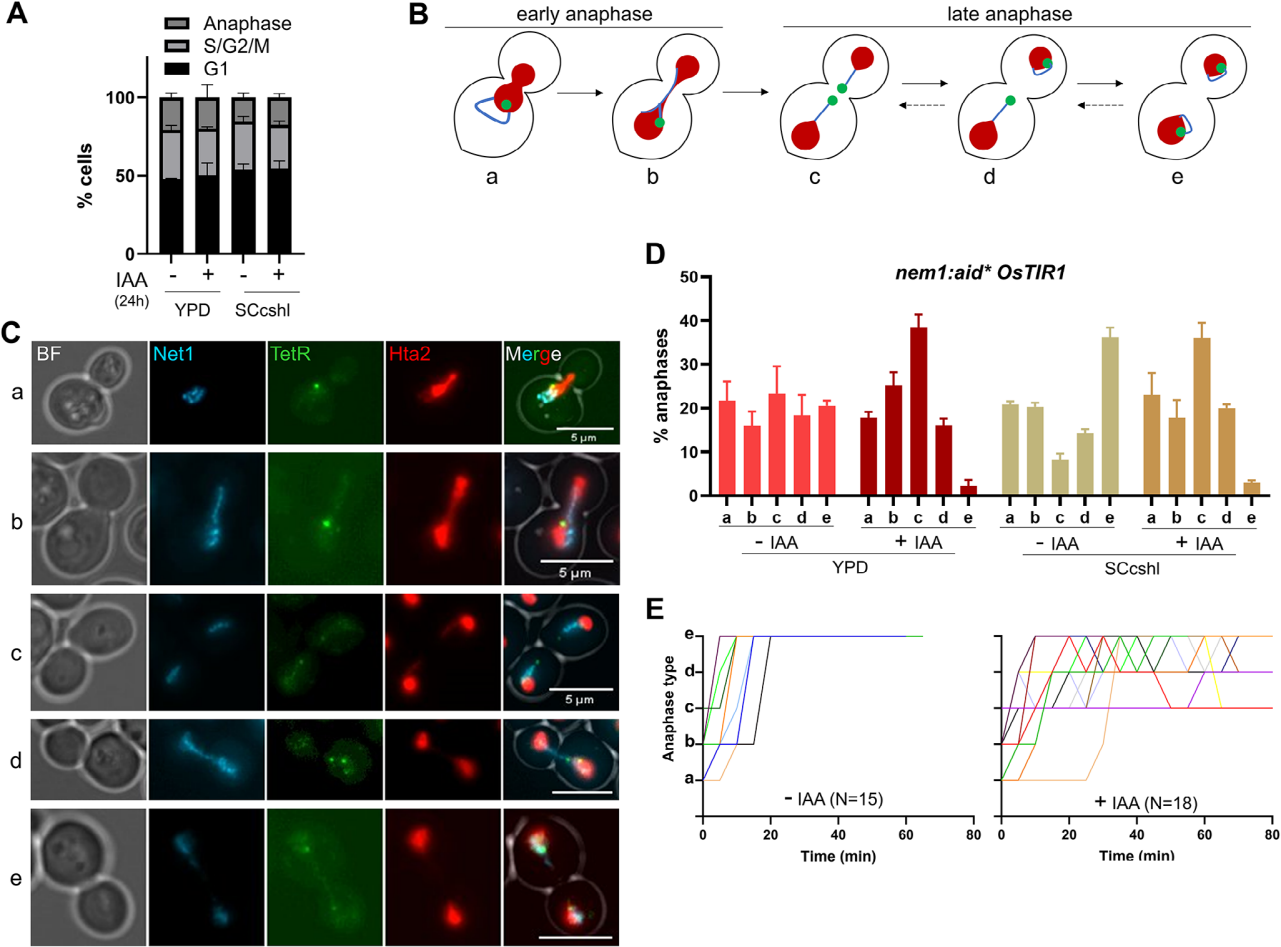
Chromosome XII and rDNA segregation patterns in cells depleted of Nem1 while growing in nutrient rich and complete minimum media. (A) Cell cycle distribution of log‐phase cultures in YPD and SCcshl with (−IAA) or without (+IAA) Nem1 (mean ± SEM, *n* = 3). (B) Schematic illustration of cXII segregation. In early anaphase (unresolved rDNA): (a) the nucleus commences its elongation through the division axis while the nucleolus/rDNA remains on one edge; (b) cXIIr resolves from centromere to telomere, which unzips the rDNA to form a bridge between mostly segregated nuclear masses. In late anaphase (segregated rDNA): (c), distal cXIIr regions are the last to be resolved and lag behind the rest of the genome; (d and e) rDNA compaction pulls distal regions to complete segregation. This latter process can occur asynchronously so that one sister cXIIr can appear in an advanced segregation state (d). (C) Representative micrographs of the drawings of (B). (D) Quantification of the different rDNA and cXIIr segregation figures (mean ± SEM, *n* = 3). Note that Nem1 depletion increases the percentage of late anaphase cells with lagging cXIIr. The growth media had little influence on this pattern (unpair *t* test for “type e” in −IAA vs. +IAA render *p* < 0.001 for both YPD and SC). (E) Transitions between the five anaphase types in the presence and absence of Nem1 (*N*, number of cells followed by time‐lapse super‐resolution microscopy; each colored line corresponds to a cell). Time zero corresponds to when the cell entered anaphase (type a).

Our experimental strategy of depleting Nem1 for just 5–7 generations (24 h with IAA), differed greatly from previous works using knock‐out *nem1* mutants, whose consequences can only be addressed after at least 40–50 generations (the generations needed to form a colony plus the ensuing overnight liquid culture). To see whether a single cell cycle was sufficient to form the protrusions, we grew the strain without IAA and then treated the log‐phase culture with 1 mM IAA for only 3 h, enough time for all cells to complete one cell cycle in YPD and SCcshl. We observed that the percentage of G1 and S/G2/M cells with protrusions after this short IAA incubation was similar to that seen after 24 h (Figure 2E), with similar rDNA shapes (Figure 2F), and only a slightly lower proportion of cXIIr‐Tel within the rDNA bar (Figure 2G). Of note, no differences were observed in the length of the protrusions between 3 and 24 h (Figure 3H).

To understand how Nem1 depletion affects mitotic progression, we examined cXIIr and rDNA segregation patterns in Nem1‐depleted cells during anaphase, which represented around 15% of cells in the YPD and SCcshl asynchronous populations (Figure 3A). As reported before (Machín et al. 2005), and also confirmed in Figure 1, cXIIr follows a predictable segregation pattern during mitosis, with early anaphase marked by unresolved rDNA and later stages showing full resolution and segregation of distal cXIIr regions. This pattern can be more precisely fine‐tuned by adding further intermediate steps under the assumption of the centromere‐to‐telomere unzipping model (Figure 3B,C). On the one hand, early anaphase can be subdivided into two stages based on whether the rDNA remains at one nuclear edge (type a) or forms a bridge across the neck (type b). On the other hand, late anaphase could be split into three categories on the basis of the recoiling of the cXIIr telomere, which is last to resolve and segregate (Machín et al. 2005). Thus, right after its late resolution, two very close spots are seen near the bud neck, with lagging threads of chromatin linking them to their corresponding daughter‐to‐be nuclear mass (type c). After that, recoiling of the cXIIr through a spindle‐independent mechanism brings the cXIIr telomeres close to the main segregated nuclear masses (type e) (Machín et al. 2005), a process that can also occur asynchronously (type d). When we compared these five anaphase phenotypes in the presence or absence of Nem1 (− vs. +IAA in the nem1‐aid* strain), we observed a marked shift from “type e” to “type c”; that is, the absence of Nem1 delayed late mitotic cells from pulling lagging cXIIr after resolution (Figures 3D and S3A,B). This IAA‐dependent shift occurs in both YPD and SCcshl for the nem1‐aid* strain, but not for *NEM1*, again disregarding IAA off‐target effects (Figure S3C). A similar decrease in “type e” anaphases was observed after depleting Nem1‐aid* for 3 h (Figure S3D). Additionally, the length of cXIIr protrusions in “type c and d” anaphases was similar after 3 and 24 h with IAA (Figure S3E), with “type d” being slightly larger than “type c.”

### 3.3 rDNA Protrusions Persist in Early Anaphase and Become Dynamic Lagging cXIIr Sisters in Late Anaphase

In early anaphase, rDNA protrusions were still seen for most cells without Nem1, indicating that they are not immediately absorbed into the nuclear mass when the NE begins its anaphase expansion (Figure 3C, type a cell; Figure S3A). However, the fate of this protrusion once the rDNA resolution begins was not clear (Figure 3C, type b cell), so we filmed asynchronous cells transiting through anaphase to better follow it (Figures S4–S6; Movies S1‐S3). In relation to the axis of nuclear elongation, two spatial configurations could be distinguished for the rDNA protrusion (Figure S7), one in the same axis of segregation (more abundant in SCcshl), and one more or less perpendicular to it (more abundant in YPD). When in the same axis, the protrusion was no longer distinguishable once the rDNA started its resolution and formed a bridge between the segregating nuclear masses (Figures S4 and S5; Movies S1 and S2). When parallel to the axis, it also transited to a bridge in most cases; however, we could still observe instances where the protrusion remained in a ∼90° angle even during the cXIIr resolution by the centromere‐to‐telomere unzipping mechanism (Figure S6; Movie S3, lower cell). Such anaphase recordings, together with the association between presence of protrusion in early anaphase and more lagging cXIIr in late anaphase, lead us to propose that the single rDNA protrusion in interphase and early anaphase becomes the two lagging cXIIr sisters in late anaphase.

Finally, a clear dynamism in the lagging cXIIr sisters could be observed in movies (Figures 3E and S6; Movie S3, upper cell), with cells reversing from “type e” to “type c” when Nem1 was depleted. This phenomenon has been described before during late mitotic arrests, and is exacerbated upon DNA damage (Ayra‐Plasencia and Machín 2019; Ivanova et al. 2020). Thus, the absence of Nem1 appears to be a third condition that promotes this dynamic behavior for segregating cXIIr sisters. In context, the most likely explanation is the persistence of the protrusion itself through anaphase, although alternative scenarios such as late DNA damage or slower mitotic exit in the absence of Nem1 should be considered. On the other hand, the persistence of types “c” and “d” in movies, with offsprings likely in the next G1 (Figure S6; Movie S3, upper cell), suggests a continuity of the protrusion through generations.

### 3.4 Junctions Between the NE and Vacuoles Are Maintained During Anaphase

**Figure 4:**
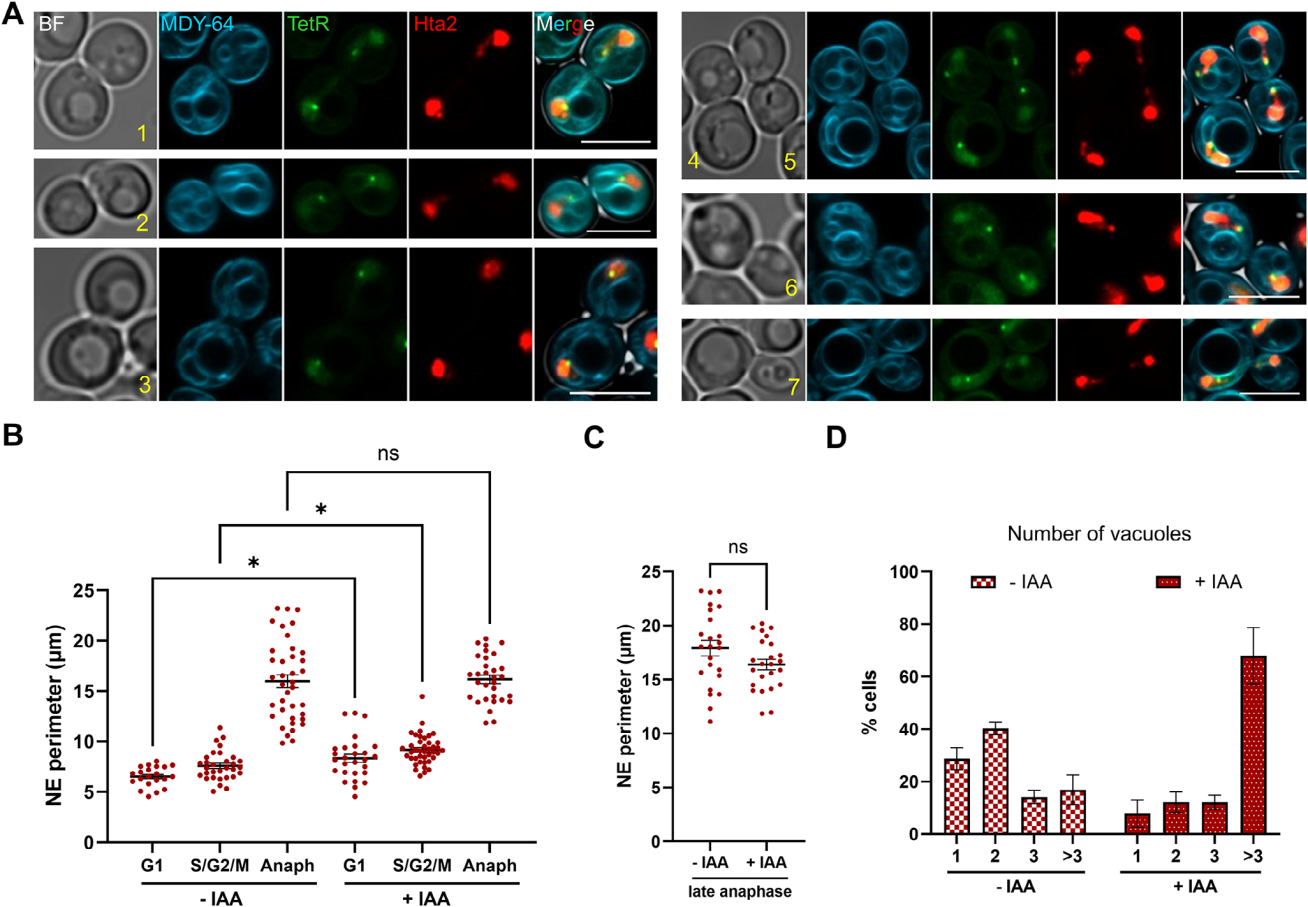
The NE‐vacuole axis in anaphase with and without Nem1. An asynchronous log‐phase culture of FM2748 was stained with MDY‐64 dye under conditions that label both the NE and the vacuolar membrane. MDY‐64 is read in the CFP channel, and its brightness overshadowed Net1, which could not be assessed here. (A) Seven representative anaphase cells. Note the close interaction between the NE and vacuoles. Lagging cXIIr often bends around or is attached to vacuole(s). (B) Single cell analysis of the NE perimeter at different cell cycle stages in the presence and absence of Nem1 (mean ± SD). One way ANOVA was applied for statistical assessment. The effect of Nem1 for each cell cycle stage was the only post hoc comparison included (*p < 0.05; ns, *p* > 0.05). (C) As in (B), but only counting late anaphase cells (i.e., elongated or binucleated Hta2 with two *tetO:1061*). The two‐tailed Student's *t* test was applied. (D) Vacuole number per cell (all cell cycle stages) with and without Nem1 (mean ± SEM, *n* = 3; unpair *t* test for “>3 vacuoles” renders a *p* = 0.0134).

Previously, we reported that the NE protrusions that arise after both prolonged G2/M blocks and in G2/M cells depleted from Nem1 are almost always in close association with vacuoles (Matos‐Perdomo et al. 2022). Morphologically, this association manifests as either small vacuoles often surrounding protrusions or large vacuoles anchoring and bending them to a great extent. The vacuoles can be stained using several specific dyes. The MDY‐64 dye can label both the NE and vacuolar membranes in blue (CFP channel), so it appropriately serves the purpose of looking at NE‐vacuole confluence. With this dye, we could observe that the intimate union between the NE and vacuoles was still present in late anaphase, regardless of the presence and absence of Nem1 and the degree of lagging cXIIr (Figure 4A). In cells with lagging cXIIr, vacuoles often shape this protrusion (cells #4‐6), or even pull the protrusion out of the main nuclear mass (cell #1). The dynamics of the relationship between vacuoles and the cXIIr protrusion were further assessed through video‐microscopy with the vital dye Blue CMAC, which stains the vacuolar lumen, confirming that NE‐vacuole ties remain in late anaphase (Figures S6; Movie S3).

Additionally, we quantified the perimeter of the MDY64‐stained NE in the presence and absence of Nem1, observing a larger perimeter in its absence for cells in G1 and S/G2/M (Figure 4B). This fulfilled the expectation of having an extended NE in Nem1‐depleted cells. However, this difference was not found in anaphase. By contrast, Nem1‐depleted cells tended to have even shorter perimeters, especially in late anaphase (late anaphase accounted by the presence of segregated cXIIr telomeres), although the differences were not statistically significant (Figure 4C). This surprising outcome could be attributed to two factors. On the one hand, the extended NE perimeter in anaphase stems greatly from the hourglass bridge, which can assimilate NE protrusions (Figures 1, 3, and S6; Movie S3). On the other hand, and perhaps more importantly, the number of vacuoles is higher in Nem1‐depleted cells (Figure 4D), making their individual size smaller and thus imposing less contorted stress than large vacuoles (Figure 4A; e.g., cells #3, #4, and #7). This increase in vacuole number is likely due to a shift in vacuole fusion and fission events toward the latter, as has been reported for lipin mutants (Sasser et al. 2012).

**Figure 5:**
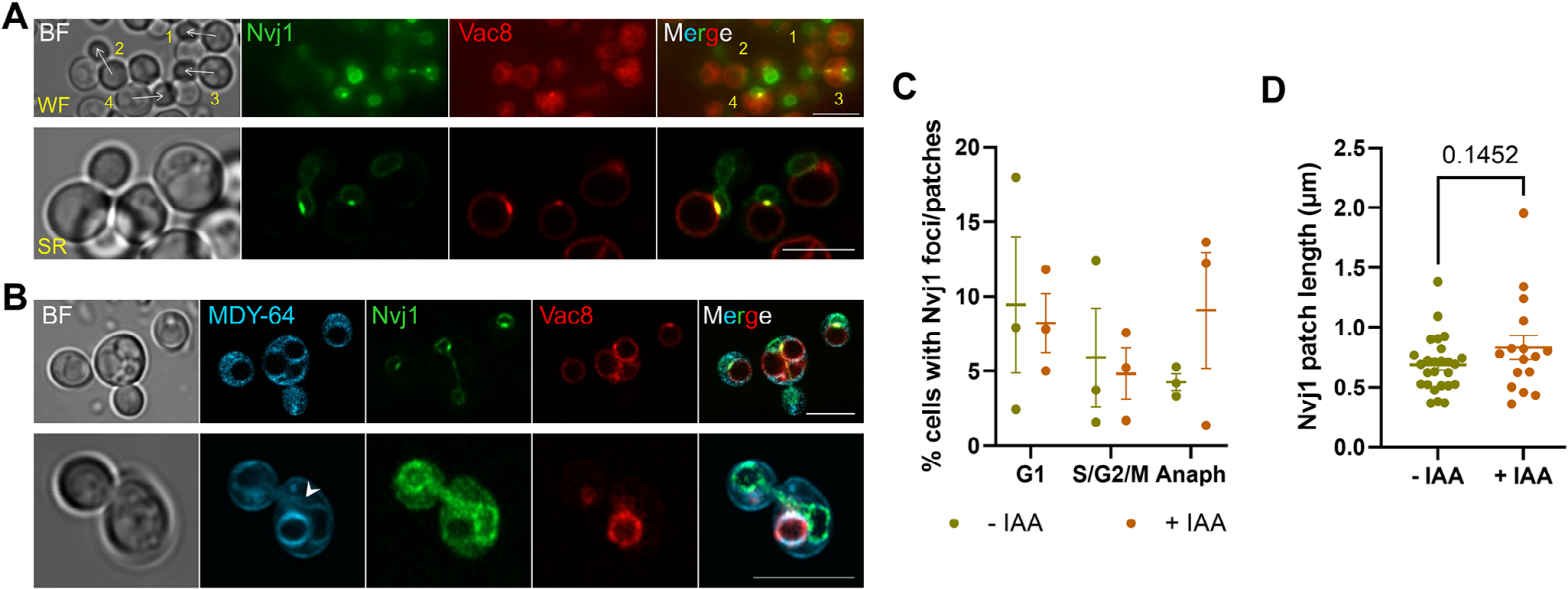
Nvj1‐Vac8 nucleus‐vacuole junctions with and without Nem1. (A) Representative log‐phase cells from a *NVJ1‐YFP VAC8‐mCherry NEM1* strain (FM2793) growing in SCcshl. The top images were taken by wide‐field fluorescence microscopy (WF), while the bottom images were taken by Airyscan 2 super‐resolution microscopy (SR). Four mitotic cells are numbered in the WF example. Note the variability of NE labelling by Nvj1. Cells #3 and #4 have Nvj1/Vac8 spots indicating NVJs. In the SR example, the cell on the left is in early anaphase and shows an Nvj1‐Vac8 patch. (B) As in (A) but with the addition of MDY‐64 dye. Both examples are from SR images and include cells in late anaphase. The upper cell contains an Nvj1‐Vac8 spot, whereas the lower cell does not show NVJ, but the NE is still tightly associated with the vacuole (white arrowhead). Scale bar corresponds to 5 µm. (C) Percentage of cells displaying Nvj1‐Vac8 spots in a NVJ1‐YFP VAC8‐mCherry nem1:aid* strain (FM3311) grown overnight in SCcshl with (−IAA) and without (+IAA) Nem1 (mean ± SEM, *n* = 3). Cells were grouped according to the cell cycle stage they were in (anaph is anaphase). (D) Length of Nvj1 patches (mean ± SD; all cells included regardless of cell cycle stage). The two‐tailed Student's *t*‐test was used to compare between the presence and absence of Nem1.

As mentioned above, the association between the NE and vacuoles in anaphase was apparently equal in the presence and absence of Nem1, at least by MDY‐64 staining (Figure 4). To confirm and better evaluate this, we constructed a new strain that allowed us to visualize the nucleus‐vacuole junction (NVJ) exerted by the partner Nvj1 (NE) and Vac8 (vacuolar membrane). This NVJ is the best studied and is linked to piecemeal microautophagy of the nucleus, by which parts of the nucleolus and its NE subdomain are pinched into vacuoles and degraded (Roberts et al. 2003). It is not lost on us that this autophagy subclass could regulate the amount of NE and thus counterbalance the accumulation of NE after Nem1 depletion, in which case more Nvj1‐Vac8 NVJs would be expected. In our strain, Vac8 clearly labeled the vacuolar membrane under all conditions (Figures 5A and S8). In contrast, Nvj1 showed greater heterogeneity in its presence and distribution in the NE. For example, the perimeter of the NE could only be visualised in less than one‐third of the cells in both widefield microscopy and confocal super‐resolution (Figures 5A and S8). In YPD, less than 1% of cells in asynchronous log‐phase cultures showed foci or patches of Nvj1‐Vac8 (indicative of NVJs). In SCcshl, this percentage was 5–10 times higher, probably reflecting lower TORC1 activity and higher levels of autophagy in this minimal growth medium. However, no differences were observed between the presence and absence of Nem1, neither in the percentage of foci/patches nor in the length of the patches (Figure 5A–C). Remarkably, Nvj1‐Vac8 NVJs were observed in early and late anaphase (Figure 5B–D), confirming that NVJs can coexist with chromosome segregation in closed mitosis. It is noteworthy that in anaphases without Nvj1‐Vac8 NVJs, but where Nvj1 uniformly labelled the NE, intimate contacts between the NE and vacuoles could still be seen (Figure 5B, arrowhead in the lower cell). Therefore, it is likely that other NVJs maintain the association between these two organelles.

## 4 Conclusions

**Figure 6:**
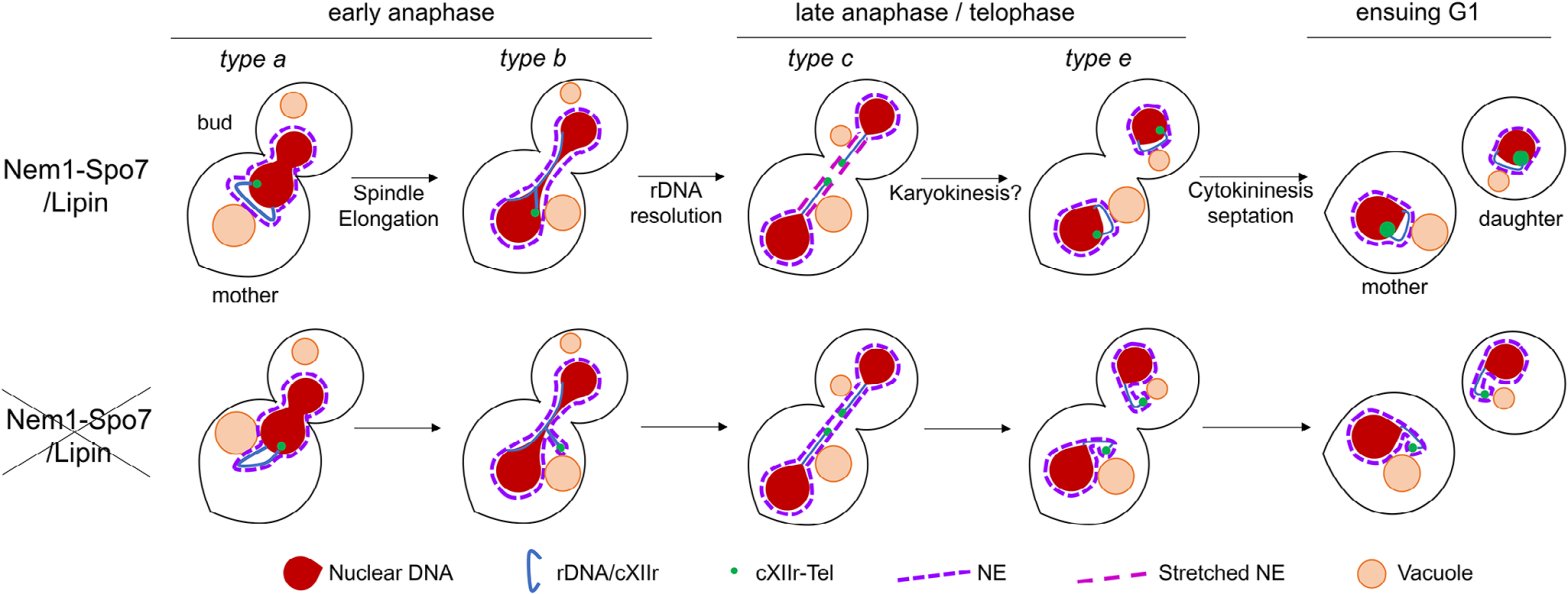
Summary and models of how an excess of NE leads to lagging chromosome arms. In the presence of Nem1 (upper diagram), the NE is maintained at a size appropriate for NE elongation in anaphase. We propose that the NE surface is short enough to exert some tension on the NE bridge of the long hourglass late anaphase nucleus, which might favor karyokinesis and stabilization of *type e* late anaphases. In the absence of Nem1 (lower diagram), the excess of NE would be absorbed by the bridge, resulting in less tension and affecting karyokinesis and NE retraction, and thus the recoiling of cXIIr (more *type c* late anaphases). This in turn would lead to the perpetuation of the rDNA/cXIIr protrusions across generations.

Closed mitosis is undertaken without dismantling the nuclear envelope (NE). Here, we have examined the closed mitosis in *S. cerevisiae* under conditions of excess NE due to Nem1 depletion. Pre‐anaphase cells are characterized by the presence of a NE evagination that contains a protruding rDNA together with other parts of the chromosome arm where the rDNA resides (chromosome XII right arm; cXIIr) (Figure 2) (Campbell et al. 2006; Matos‐Perdomo et al. 2022). We have observed that nuclear division proceeds efficiently in the absence of Nem1, while the protruding rDNA/cXIIr is converted into lagging cXIIr sisters by the end of anaphase (Figure 3). This lagging rDNA/cXIIr phenotype persists long enough to explain its presence as protrusions in G1 cells (Figure 2; Figure S6 and Movie S3). We propose a model in which the excess of NE in Nem1‐depleted G2/M cells is deposited in the division bridge by late anaphase (Figure 6), a scenario supported by our live‐cell imaging experiments (Figures S4–S6 and Movies S1–S3) and the fact that the rDNA/cXIIr is last to segregate (Figure 3) (Campbell et al. 2006; Machín et al. 2005). Such an excess would lead to a less tensioned NE bridge, which would in turn make the bridge less prone to retract toward the segregated DNA masses once karyokinesis is completed (Figure 6), propagating the protrusions through generations. In an alternative, yet not mutually exclusive scenario, the presence of anaphase NE‐vacuole junctions (NVJ) around the rDNA could anchor such NE excess out of the main nuclear body (Figures 4 and 5). However, NVJs associated with piecemeal microautophagy of the nucleus are not more abundant after Nem1 depletion, suggesting that this process does not counterbalance the excess of NE.

Altogether, our findings highlight that the NE is not a passive bystander during closed mitosis; rather, a balanced size of the NE in anaphase actively contributes to ensuring the timely segregation of chromosomes and proper spatial distribution of chromatin in ensuing cell cycles.

## Author Contributions

**Noelia Rodríguez‐Herrera**: methodology, investigation, formal analysis, visualization. **Silvia Santana‐Sosa**: methodology, investigation, visualization. **Sara Medina‐Suárez**: methodology. **Samantha Morais‐Armas**: formal analysis. **Emiliano Matos‐Perdomo**: methodology, writing–review and editing. **Félix Machín**: conceptualization, formal analysis, visualization, resources, supervision, project administration, funding acquisition, curation, writing–original draft, writing–review and editing.

## Conflicts of Interest

The authors declare no conflicts of interest.

## Supporting information



Supporting Information

Supporting Information

Supporting Information

Supporting Information

## Data Availability

All experimental data are included in the article and supplementary material. Yeast strains are available upon request.
