# Supplementary material for: Chromosome Segregation in Closed Mitosis Under an Excess of Nuclear Envelope
Source: Biol Cell. 2025 May 20;117(5):e70011. doi: 10.1111/boc.70011 (PMC12090705; doi:10.1111/boc.70011)
Supplement: Supplementary file 1 — Supporting Information [file BOC-117-e70011-s003.pdf]

**Chromosome segregation in closed mitosis under an excess of nuclear envelope**

Noelia Rodríguez-Herrera[1,2], Silvia Santana-Sosa[1,2], Sara Medina-Suárez[1,2], Samantha Morais-Armas[1,2], Emiliano Matos-Perdomo[1,#], and Félix Machín[1,2,3,*]

[1] *Unidad de Investigación, Hospital Universitario Nuestra Señora de Candelaria, Instituto de Investigación Sanitaria de Canarias (IISC), 38010 Santa Cruz de Tenerife, Spain.*

[2] *Instituto de Tecnologías Biomédicas, Universidad de La Laguna, 38200 San Cristóbal de La Laguna, Spain.*

[3] *Facultad de Ciencias de la Salud, Universidad Fernando Pessoa Canarias, 35450 Las Palmas de Gran Canaria, Spain.*

[#] *Present address: Centre for Chromosome Biology, University of Galway, H91 W2TY Galway. Ireland*

* Corresponding author. *Unidad de Investigación, Hospital Universitario Nuestra Señora de Candelaria, Ctra del Rosario 145, 38010 Santa Cruz de Tenerife, Spain.*

*E-mail address*: fmachin@fciisc.es (F. Machín).

SUPPLEMENTARY MATERIAL

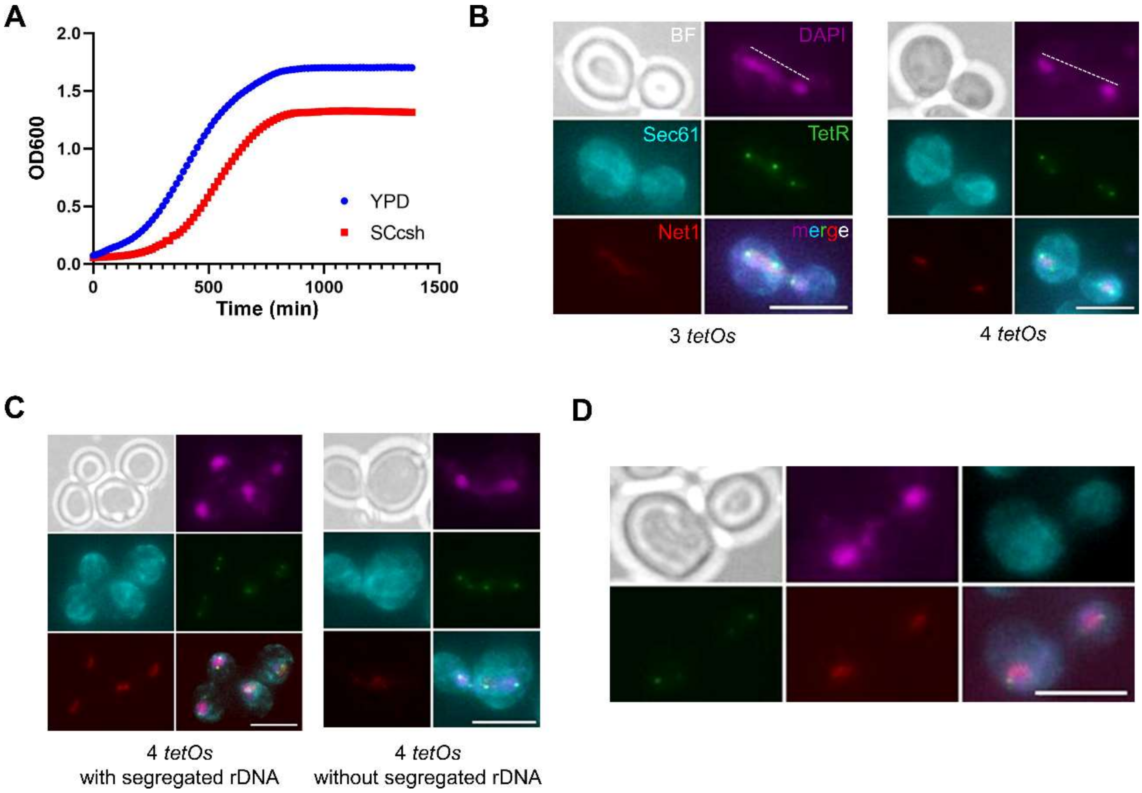


**Figure S1. Segregation patterns of rDNA/cXIIr in asynchronous cultures.** This Figure is related to Figure 1. **(A)** Growth curves of the wild type FM2658 strain in YPD and SCcshl. The strain was first grown on SCcshl plates for 3 days, and a small patch was resuspended in water and then inoculated into the corresponding media to yield an initial inoculum of 0.1 OD600. Real time growth was followed in a Tecan Spark incubator. Note that growth in SCcshl is slower. **(B)** Representative pictures of early (3 *tetOs*) and late (4 *tetOs*) anaphases. The dashed white line represents the measured length of the elongated DAPI-stained nuclear DNA mass. Quantifications in Figure 1C and D. **(C)** Representative pictures of late (4 *tetOs*) anaphases with and without rDNA resolution. Quantifications in Figure 1F. **(D)** Representative picture of a DAPI bridge in a late (4 *tetOs*) anaphase with segregated rDNA. Quantifications in Figure 1G. Scale bars correspond to 5 µm.

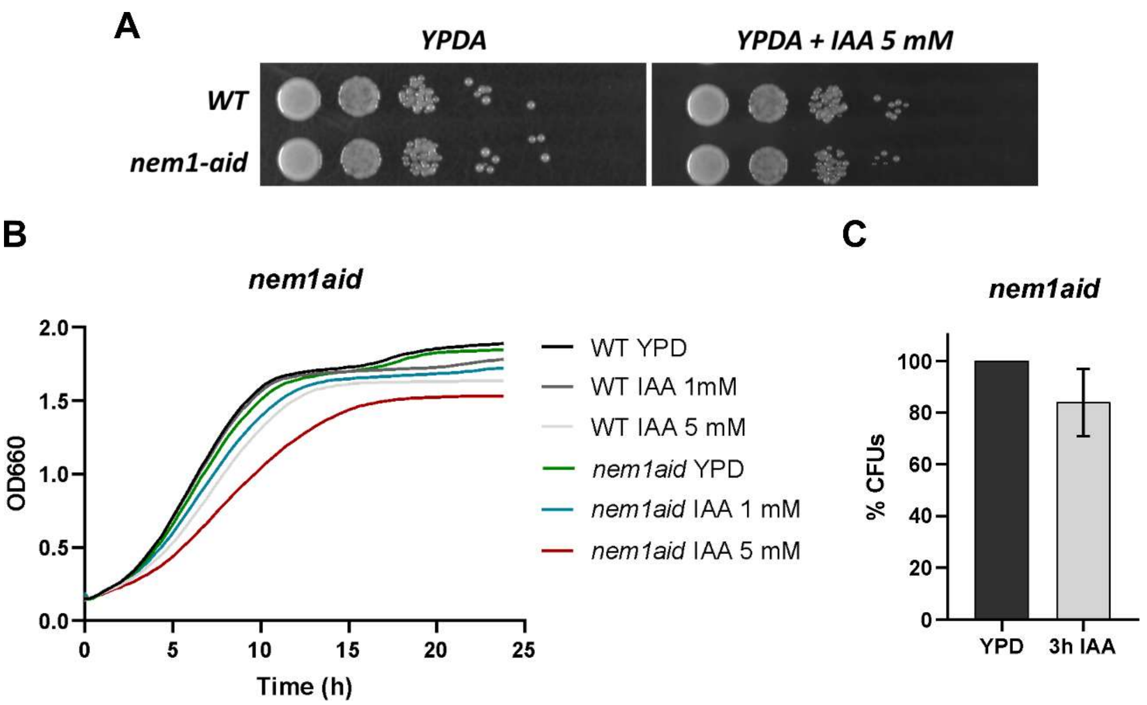


**Figure S2. Fitness of Nem1 depletion.** This figure is related to Figure 2. **(A)** Real time growth curves of FM2748 (*nem1:aid OsTIR1)* and its wild type (WT) parent FM2707 (like FM2748 but with a wild type *NEM1*). Growth was assessed in nutrient-rich YPD medium without IAA and with 1 mM or 5 mM IAA. **(B)** Spot assay of WT and *nem1:aid* strains on YPD and YPD plus 5 mM IAA plates. **(C)** Clonogenic assay of *nem1:aid* after 3h incubation with 5 mM IAA (mean ± sem, n=2). Survival (% CFU, colony forming units) was normalized to a mock treatment.

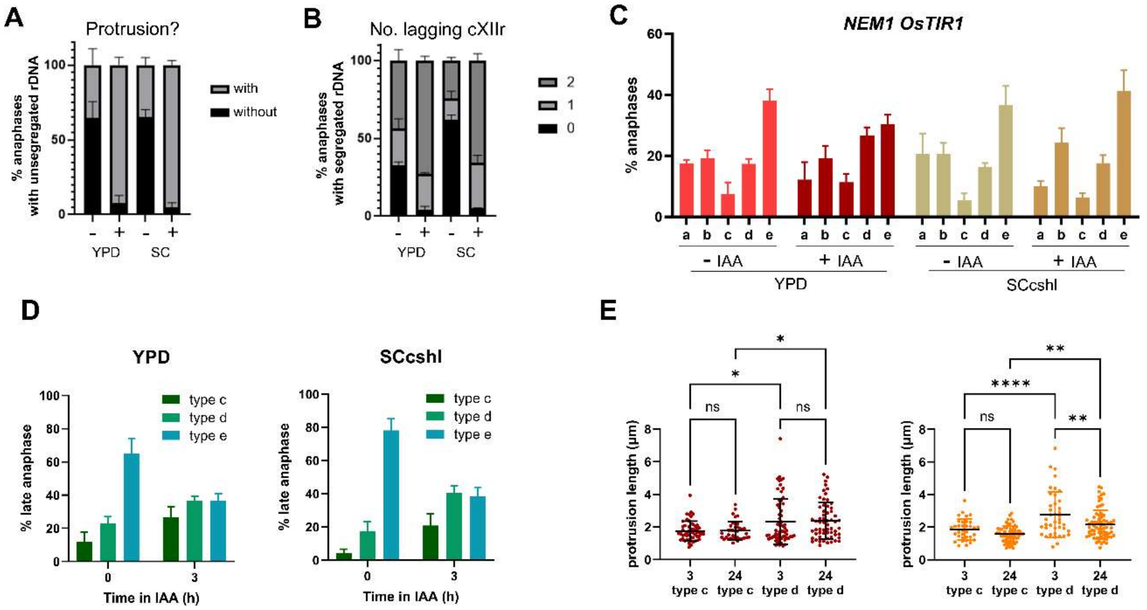


**Figure S3. Chromatin protrusions in anaphase cells with and without Nem1.** This figure is related to Figure 3. **(A)** Presence of protrusions in early anaphase cells in which the rDNA has not yet been segregated (type a and b cells in Figure 3B and C); **(B)** Number of lagging cXIIr sisters in late anaphase cells where the rDNA has already been segregated (type c to e cells in Figure 3B and C). Protrusions as seen by Hta2. Lagging cXIIr as seen by Hta2 plus *tetO* at the tip. Note that lagging cXIIr could be considered a kind of protrusion; - or + denotes incubation without or with IAA (presence and absence of Nem1). **(C)** Analysis of early (a,b) and late (c,d,e) anaphase subtypes in the *NEM1* strain after overnight growth in the indicated growth media and IAA conditions. This is the control for the experiment shown in Figure 3D for the *nem1:aid** strain (mean ± sem, n=3). **(D)** Late anaphase subtypes in the *nem1:aid** strain before and 3h after IAA addition (mean ± sem, n=3). **(E)** Length of lagging cXIIr after 3h and 24h in IAA (mean ± SD). Type c and d were evaluated separately. One way ANOVA followed by the Tukey test was applied for statistical assessment (****, p<0.0001;**, p<0.01; *, p<0.05; ns, p>0.05).

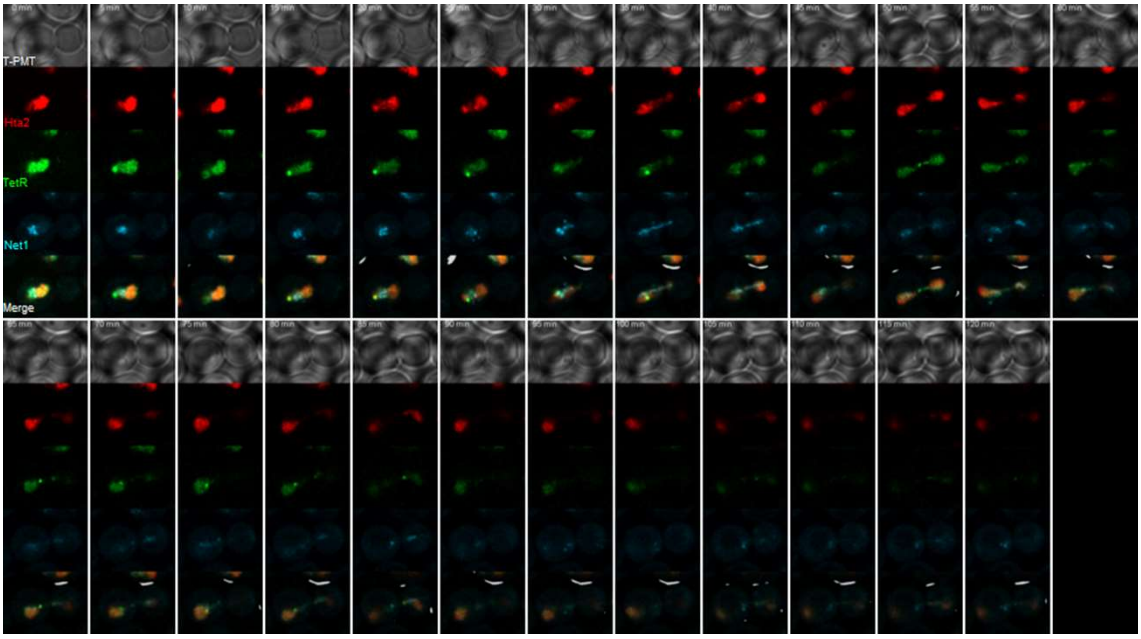


**Figure S4. Time lapse of rDNA and chromosome XII segregation in the absence of Nem1 (cell example 1).** This figure is related to Figure 3. This time lapse corresponds to Movie S1. The video-microscopy was recorded on the surface of Lab-Tek II wells with Airyscan II superresolution confocal microscopy. Cells were grown in SCcshl broth and attached to the surface with concanavalin A before filming. In this first example, the cell was in G2/M at the start of the recording (0'), and had an rDNA protrusion with the cXIIr telomere (green spot) at the tip (5'-25'). Early anaphase spanned just 10' (from 30' to 40'), and the elongating chromatin (Hta2) with the rDNA bridge, which segregated last, can be easily followed. By minute 50, the cell is in late anaphase. The cXIIr sister telomere on the right is lagging behind for most of the rest of the movie (50'-85'). Photobleaching from min 90 onwards precluded further analysis.

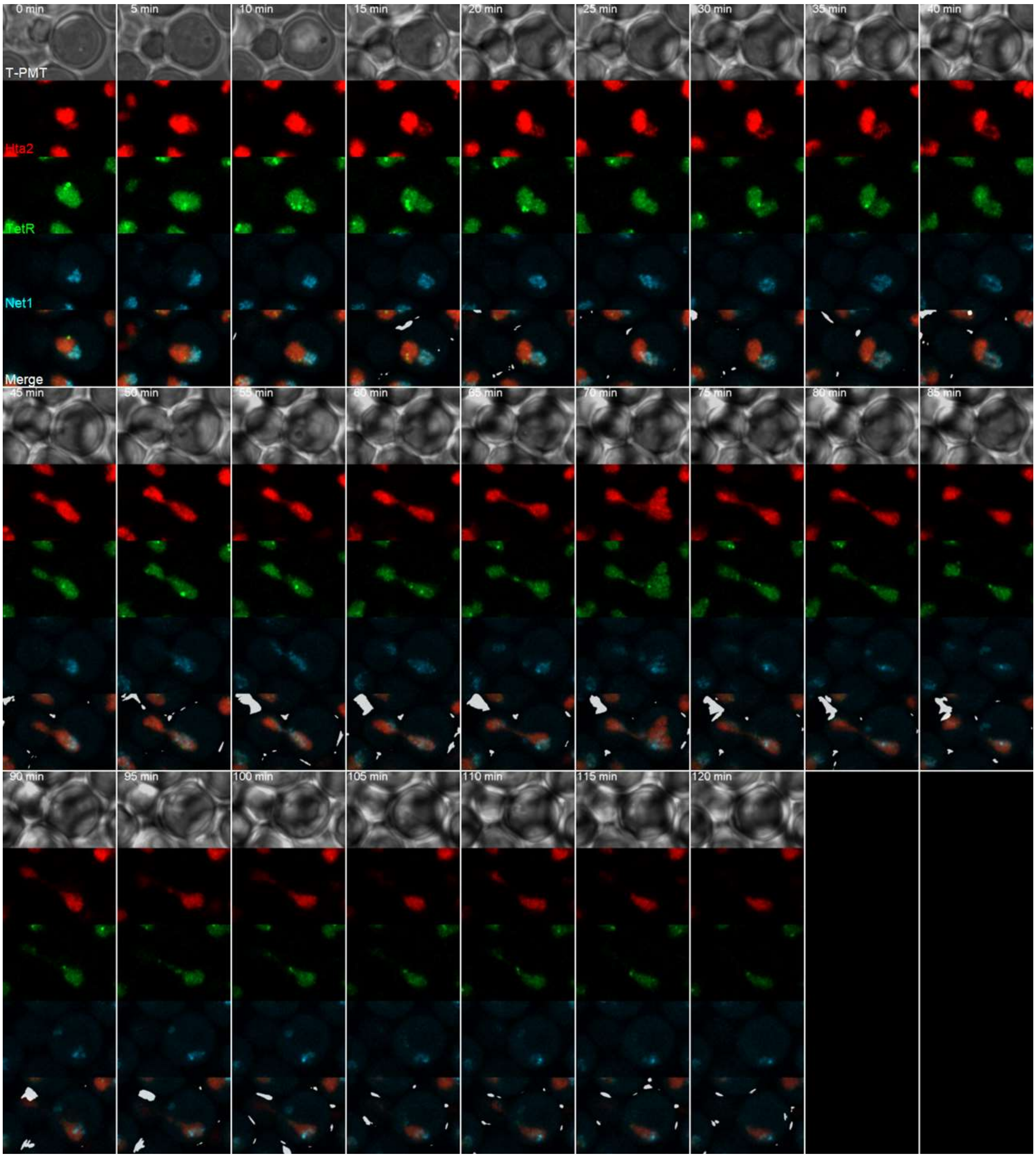


**Figure S5. Time lapse of rDNA and chromosome XII segregation in the absence of Nem1 (cell example 2).** This figure is related to Figure 3. This time lapse corresponds to Movie S2. The video-microscopy was recorded as in Figure S3. In this second example, the cell was in S phase at the beginning of the recording (0’), and acquired an rDNA protrusion in the form of a loop after entering G2/M (10’-15’), with the cXIIr telomere (green spot) within the main Hta2 mass. The cell spent half an hour in G2/M (15’-40’), with a bilobed nucleoplasm (TetR) caused by the expanding rDNA loop. Anaphase onset took place at frame 45’. Early anaphase spanned around ten minutes, and by frame 60’ the sister cXIIr were already resolved. However, they were endorsed in a back-and-forth movement, while a Hta2 thin bridge was also present, at least until frame 95’.

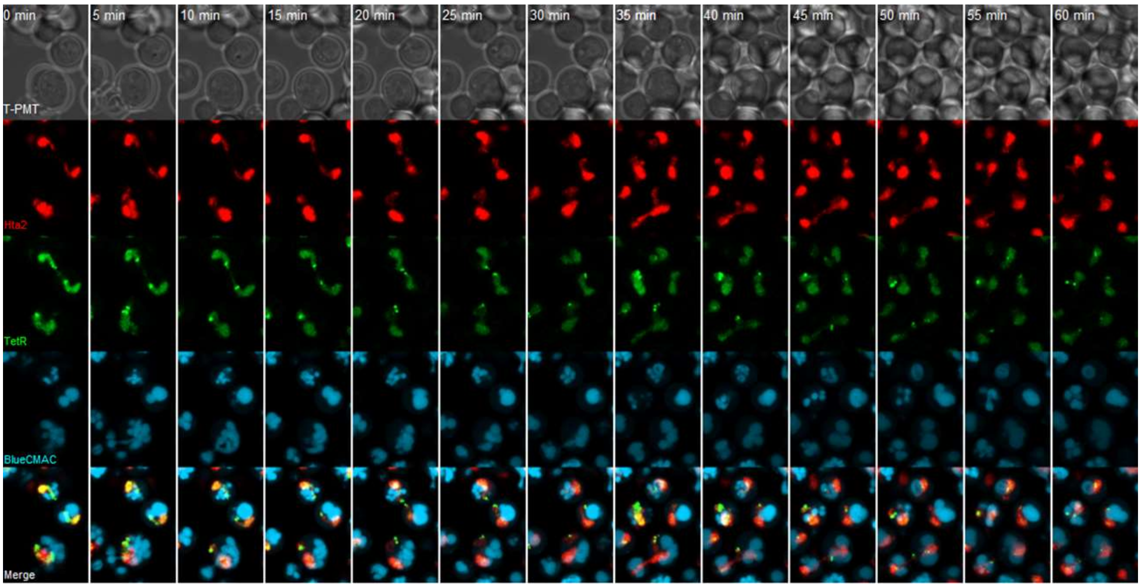


**Figure S6. Time lapse of cXIIr-vacuole interplay during anaphase in the absence of Nem1.** This figure is related to Figures 3 and 4. This time lapse corresponds to Movie S3. The video-microscopy was recorded as in Figure S3, except for the incubation with the vacuolar lumen dye Blue CMAC. The example contains two cells. The upper cell was already in late anaphase at time 0'; the lower cell was in S/G2 as inferred by the bud size. The upper cell had two cXIIr protrusions, one for each late anaphase nucleus. For the first 25 minutes, the upper cell appears to remain in late mitosis and even an approximation between the sister cXIIr telomeres is observed. From frame 30' onwards, mitosis appears to have been completed since there is a change in the orientation of the nuclear protrusions. Thus, the two new cells are in G1, yet still conserved the protrusion in the shape and size of the previous mitosis. Note how vacuoles tightly associate with protrusions, defining their shape and/or orientation. In the lower cell, a cXIIr protrusion was already present at time 0' and increased in size as the cell entered G2/M (15'-30'), making the nucleus acquire a bilobed shape. Anaphase started at frame 35' and, interestingly, was perpendicular to the protrusion axis, which allowed us to visualize how it was reabsorbed into the lagging cXIIr sisters (35'-45').

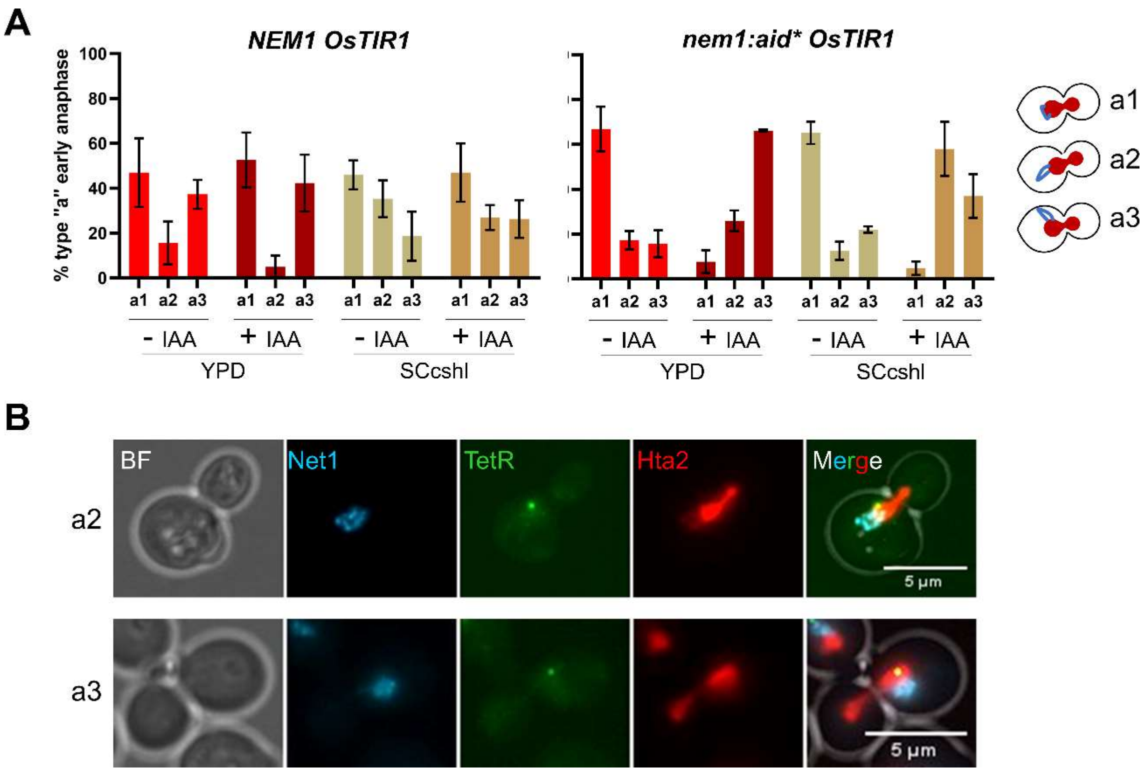


**Figure S7. Axis of rDNA protrusion relative to the segregation axis.** This figure is related to Figure 3. Only type "a" early anaphases are considered, irrespective of the position of the cXIIr telomere; a1, no protrusion; a2, protrusion follows the segregation axis; a3, protrusion is perpendicular to segregation. **(A)** Quantification of type a subclasses in the *NEM1* and *nem1:aid** strains (mean ± sem, n=3). **(B)** Representative micrographs of early anaphase protrusions of subclasses a2 and a3.

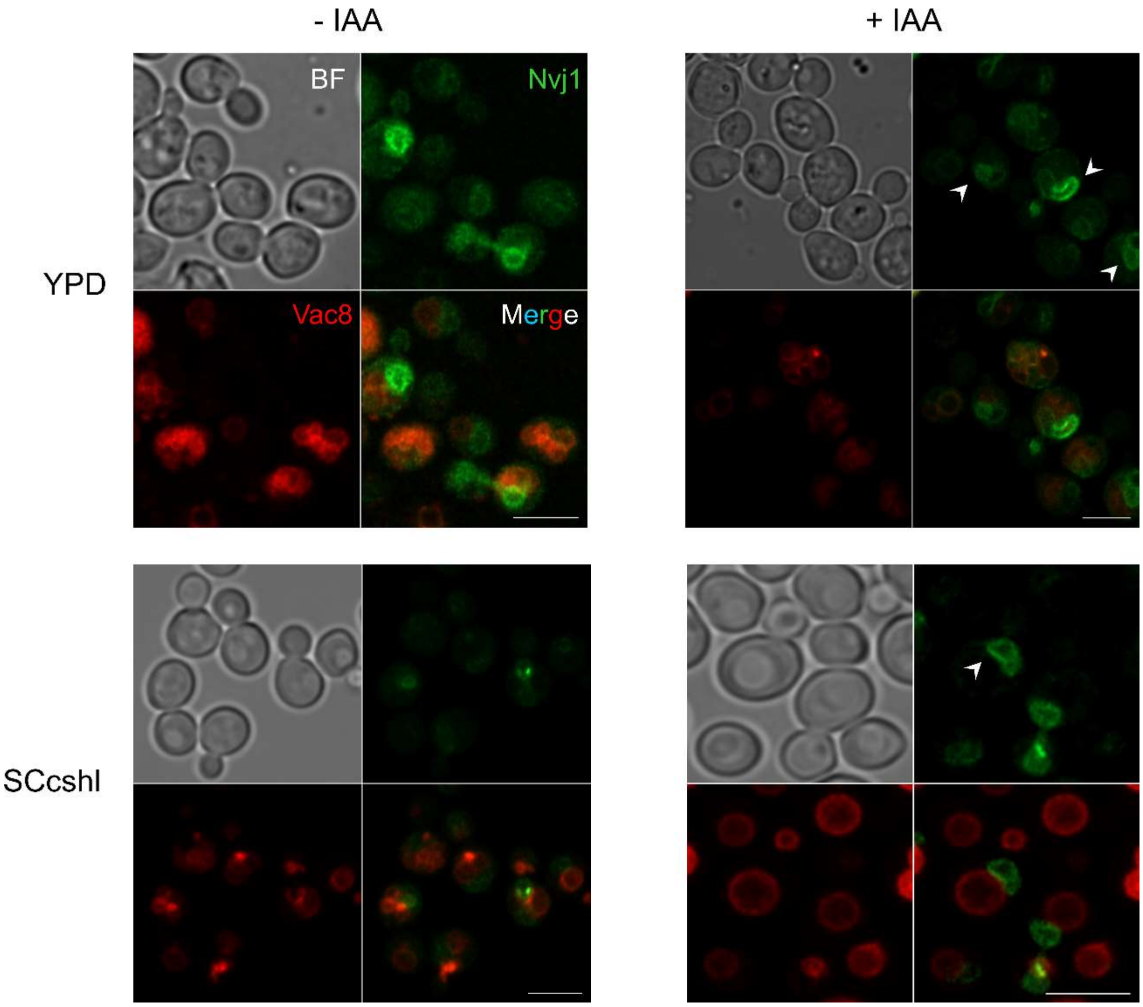


**Figure S8. Representative micrographs of the *NVJ1-YFP VAC8-mCherry nem1:aid** strain (FM3311) under the different experimental conditions (YPD vs. SCcshl and – vs. + IAA).** This figure is related to Figure 5. White arrowheads point to flares, lobes and other NE abnormalities that occur after Nem1 depletion. Scale bars correspond to 5 µm.

**Table S1. Strains used in this work.**

| Name | Genotype[1] | Origin | Use |
|---|---|---|---|
| FM2658 | YPH499 *bar1; ade2-1::TetR:YFP::ADE2; cXII(450)::tetOs::URA3; cXII(1061)::tetOs::HIS3; SEC61:eCFP::kanMX4; NET1:mCherry::natNT2* | Machín lab | Figs 1; S1 |
| FM2707 | YPH499 *bar1; ade2-1::TetR:YFP::ADE2; cXII(1061)::tetOs::HIS3; cdc15-2:9myc::Hph; ura3::ADH1p:OsTIR1:9myc::URA3; HTA2:mCherry::natNT2; NET1:eCFP::klTRP1* | Machín lab | Figs 2; S2; S3C; S7A |
| FM2748 | FM2707; *nem1:aid*:9myc::KanMX* | Machín lab | Figs 2-4; S2-S7 |
| FM2793 | YPH499 *bar1; cdc15-2:9myc::Hph; NVJ1:eYFP::kanMX4; VAC8:mCherry::natNT2* | This study | Figs 5A,B |
| FM3311 | FM2793; *ura3::ADH1p:OsTIR1:9myc::URA3; nem1:aid*:9myc::HIS3* | This study | Figs 5C,D; S8 |

[1] Semicolon (";") separates genetic modifications accomplished sequentially through transformation. Intermediate strains are omitted.

Supplemental Movie Legends.

**Movie S1. Video-microscopy of rDNA and chromosome XII segregation in the absence of Nem1 (cell example 1).** This movie is related to Figure 3. See Figure S3 for details.

**Movie S2. Video-microscopy of rDNA and chromosome XII segregation in the absence of Nem1 (cell example 2).** This movie is related to Figure 3. See Figure S4 for details.

**Movie S3. Time lapse of cXIIr-vacuole interplay during anaphase in the absence of Nem1.** This movie is related to Figure 4. See Figure S5 for details.
